# NLRP3 inflammasome inhibitor MCC950 can reduce the damage of pancreatic and intestinal barrier function in mice with acute pancreatitis

**DOI:** 10.1590/acb370706

**Published:** 2022-10-28

**Authors:** Yanghui Shen, Huobao Yang, Dansen Wu, Hangmei Yang, Donghuang Hong

**Affiliations:** 1 MM. Fujian Medical University –Shengli Clinical Medical College – Department of Critical Care Medicine – Fuzhou, China; 2 MM. Fujian Provincial Hospital – Department of Critical Care Medicine – Fuzhou, China.; 3 MD. Fujian Medical University – Shengli Clinical Medical College – Department of Critical Care Medicine – Fuzhou, China.

**Keywords:** Acute Pancreatitis, NLR Family, Pyrin Domain-Containing 3 Protein, Intestine, Small, Models, Animal

## Abstract

**Purpose:**

Abnormal activation of NOD-like receptor protein 3 (NLRP3) inflammasome can lead to the occurrence and progression of acute pancreatitis. This study investigated the protective effect of MCC950 on pancreatitis mice.

**Methods:**

Eighteen mice were randomly divided into control group, severe acute pancreatitis (SAP) group and SAP+MCC950 group. Serum interleukin (IL)-1β, IL-6 and tumor necrosis factor-α (TNF-α) were measured by ELISA. Hematoxylin and eosin (HE) staining was used to evaluate the pathological damage. Western blotting was used to detect the expression of NLRP3 inflammasome and tight junction proteins in the small intestine and pancreas.

**Results:**

MCC950 could reduce the levels of IL-6 and IL-1β in SAP mice. After treatment with MCC950, the expression levels of NLRP3 inflammasome in the pancreas of SAP mice were significantly reduced and the pathological damage to the pancreas and intestine was alleviated. Compared with the control group, the expression of tight junction protein (ZO-1,occludin and claudin-4) in the intestinal mucosa of SAP mice was decreased, and the expression of claudin-4 and occludin were upregulated after MCC950 treatment.

**Conclusions:**

MCC950 can inhibit NLRP3 inflammasome activation and significantly reduce the inflammatory response and delay the process of pancreatitis. It has therapeutic potential in the treatment of acute pancreatitis.

## Introduction

Acute pancreatitis is one of the most common diseases in the digestive system, and the incidence has increasing in recent years. The mortality of severe acute pancreatitis (SAP) can reach 32%^1^. Acute gastrointestinal injury and intestinal barrier dysfunction often occur in patients with SAP^2^, and these complications will aggravate the severity of acute pancreatitis. Thus, reducing the degree of intestinal barrier damage has become an important goal in SAP treatment^3^^,^ 4. Although current studies suggest that inflammation is involved in the progression of pancreatitis, the mechanism has not been elucidated.

Acute pancreatitis is an uncontrolled and self-destructive systemic inflammatory process that may lead to systemic inflammatory response syndrome and multiple organ failure^5^. Inflammasome cascade is an important signal pathway involved in the pathogenesis of acute pancreatitis. NOD-like receptors are intracellular pattern recognition molecules, which are important proteins involved in the inflammatory storm. NOD-like receptor protein 3 (NLRP3) is widely expressed in immune cells (neutrophils, dendritic cells, macrophages) and plays an important role in the formation of NLRP3 inflammasome^6^^,^ 7. NLRP3 inflammasome is an inflammatory complex composed of NLRP3, apoptosis associated speck like protein containing card (ASC) and cysteinyl aspartate specific protein-1 (caspase-1)^8^. It has been found that inflammasomes lead to the maturation of interleukin-1β (IL-1β)^9^. IL-1β is closely related to SAP, and its upregulation is positively correlated with the severity of the disease. After blocking the expression of IL-1β in SAP, the pathological injury and inflammation of the pancreas have been significantly reduced^10^. However, there is still no clear evidence as to whether blocking the activation pathway of NLRP3 inflammasome could reduce the damage of pancreas and intestinal barrier.

MCC950 is an effective highly specific small molecule inhibitor, which can inhibit the typical and atypical activation of NLRP3 inflammasome^11^. It has been reported that MCC950 has been successfully applied to animal models of myocardial infarction, colitis, cognitive impairment, pneumonia, and spinal cord injury^12^^-^ 16. However, the potential role of MCC950 in acute pancreatitis and intestinal injury is unclear. Hence, we decided to investigate the anti-inflammatory effect and potential regulatory mechanism of MCC950 in cerulein-induced SAP mice and analyze the protective effect of MCC950 on the intestinal barrier.

## Methods

This study protocol was reviewed and approved by the Animal Ethics Committee of Fujian Provincial Hospital, approval number No. a20191122011. All animal experiments complied with the ARRIVE guidelines and were carried out in accordance with the National Research Council’s Guide for the Care and Use of Laboratory Animals.

### Reagents

The amylase (MM-1018M1) and lipase (MM-1157M1) enzyme-linked immunosorbent assay (ELISA) kits were purchased from Jiangsu MMBio Industry Co., Ltd (Yancheng, China). Tumor necrosis factor-α (TNF-α) (KE10002), IL-1β (KE10003) and IL-6 (KE10007) ELISA kits were purchased from Proteintech Group. Primary antibodies against the following proteins were purchased: rabbit anti-occludin (df7504, Affinity, 1/1000), rabbit anti-ZO-1 (af5145, Affinity, 1/500), rabbit anti-claudin-4 (af5350, Affinity, 1/500), rabbit anti-ASC (bs-6741R, Bioss, 1/500), rabbit anti-NLRP3 (bs-10021R, Bioss, 1/500) and rabbit anti-caspase1 (22915-1-ap, Proteintech, 1/500).

### Animal treatments

Sample size calculations were performed after a pre-experiment with pancreatic tissue NLRP3 inflammasome expression levels as the outcome with four C57BL/6 mice per group. The calculated sample size was six mice in each group, with a precision of 0.05, 80% power and 95% confidence interval. C57BL/6 male mice (7 to 8 weeks old, weight 20 ± 2 g), were purchased from Henan Sikes Biotechnology Co., Ltd (SCXK, Yu, 2020-0005). The mice were randomly divided into three groups (n = 6 in each group): control group, SAP group and SAP+MCC950 group. The experiment began after the mice adapted to the environment. Acute pancreatitis mice model was induced by cerulein hyperstimulation. Cerulein solution (5 μg/mL) was administered by intraperitoneal injection at the dose of 10 mL/kg, once per hour and 12 times in total. During the last injection of cerulein, the SAP mice model was also induced by intraperitoneal injection of lipopolysaccharide (1 mg/mL) solution at the dose of 10 mg/kg. In the SAP+MCC950 group, MCC950 solution (5 mg/mL) was injected intraperitoneally at the same time as the first shot of cerulein. The dosing regimen for MCC950 was determined based on pharmacokinetic parameters and with reference to the study by Coll *et al.* 17. Each mouse in the control group was injected 10 mL/kg intraperitoneally with normal saline every hour, for a total of 12 times.

### Sample collection

Twenty-four hours after the first administration, mice were deeply anesthetized with sodium pentobarbital at the dose of 0.12 g/kg. After anesthesia, the chest cavity of mouse was opened and blood samples were collected from the heart. The blood samples were centrifuged, and the supernatants were taken for ELISA detection. The pancreas and small intestine were taken for follow-up western blotting and hematoxylin and eosin (HE) staining.

### ELISA

The double-antibody sandwich method was used to determine the contents of amylase, lipase, C-reactive protein (CRP) and D-lactic acid in serum, as well as the levels of IL-6, IL-1β and TNF-α. The operation was carried out according to the instructions of the ELISA kit.

### Histology

Fresh pancreas and small intestine specimens were fixed overnight with 4% formaldehyde, rinsed with tap water for 2 h, ethanol gradient dehydration, paraffin embedding, and sliced into 5-μm thickness. The slices were baked, dewaxed in xylene, hydrated by upgraded ethanol solution, and stained with HE. Pancreatic and small intestinal injuries were evaluated at 100× magnification under an optical microscope (BX43, Olympus). The histological evaluation of the pancreas was determined by edema, hemorrhage, inflammatory cell infiltration and acinar cell necrosis^18^. The histological evaluation of the small intestine is based on the damage and abscission of villi, interstitial edema, and mesothelial cell space^19^. The specific scoring criteria are shown in Tables 1 and 2. The histological scores were assessed by two professional pathologists blinded to the study groups.

**Table 1:** Pathological scoring criteria for pancreas.

Score	Edema	Hemorrhage	Inflammatory cell infiltration	Necrosis
0	No	No	0-1/HP	No
1	Mild leaf gap widening	Yes	2-10/HP	Necrotic area 1%–10%
2	Severe leaf gap widening		11-20/HP	Necrotic area 11%–20%
3	Acinar gap widening		21-30/HP	Necrotic area 21%–30%
4	Cell gap widening			Necrotic area >30%

**Table 2:** Pathological scoring criteria for small intestine.

Score	Mucosal Damage
0	Normal mucosal villi.
1	Subepithelial Gruenhagen’s space developed and usually at the apex of the villus; often with capillary congestion.
2	Extension of the subepithelial space with moderate lifting of epithelial layer from the lamina propria, also with central chylous duct dilatation.
3	Villi fall and a few tips may be denuded. The lamina propria is markedly edematous.
4	Denuded villi with lamina propria and dilated capillaries exposed. Epithelial cell denaturation and necrosis. Increased cellularity of lamina propria may be noted.
5	Villi loss with digestion and disintegration of lamina propria; hemorrhage and ulceration.

### Western blotting

Total protein was extracted from the pancreas and small intestine tissues using standard procedures after recovery from −80 °C storage. BCA assay (CW0014s, Kangwei Century) was used to detect protein concentration. The proteins were separated by sodium dodecyl sulfate-polyacrylamide gel electrophoresis (SDS-PAGE) and transferred to the polyvinylidene fluoride membrane. The membranes were blocked with 3% skimmed milk and incubated overnight with primary antibodies: NLRP3, ASC, caspase-1, ZO-1, occludin, claudin-4, separately. HRP-labeled goat anti-rabbit IgG (H+L) was used as the secondary antibody. The enhanced chemiluminescence luminescent solution was evenly dropped on the polyvinylidene fluoride membrane for 1 min and developed in the Odyssey FC imaging system. The signal intensity of protein expression bands was calculated by ImageJ software. Beta-actin was used as the protein loading control for Western blot analysis.

### Statistical analysis

All data were statistically analyzed by SPSS 26.0 and GraphPad Prism 7. Continuous variables were summarized as mean (± standard deviation) for normally distributed variables. The significant difference between the groups was analyzed by one-way ANOVA with the Fisher’s least significant difference test for post-hoc comparison between groups. Values with p < 0.05 were considered statistical significance.

## Result

### The expression of serum cytokines in SAP mice

**Figure 1:**
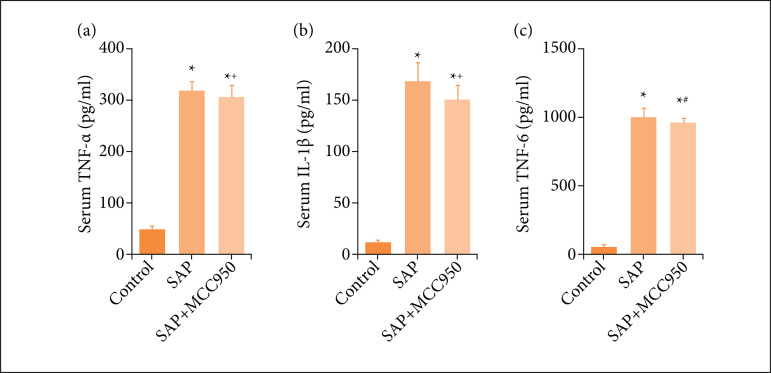
Expression of serum inflammatory factors (TNF-α, IL-1β and IL-6); **(a)** TNF-α; **(b)** IL-β; **(c)** IL-6. N = 6; ^*^ p < 0.001 vs. control; ^+^ p < 0.05 vs. SAP; ^#^ p < 0.01 vs. SAP.

Compared with the control group, the expression levels of IL-6, IL-1β and TNF-α in serum of the SAP group were significantly higher (Fig. 1). After intraperitoneal injection of MCC950, the expression of serum IL-6 and IL-1β decreased (Fig. 1b,c). For TNF-α, there was no significant difference between SAP and MCC950 treated groups (Fig. 1a).

### Effects of MCC950 on the expression of NLRP3 inflammasome in pancreas and small intestine

**Figure 2:**
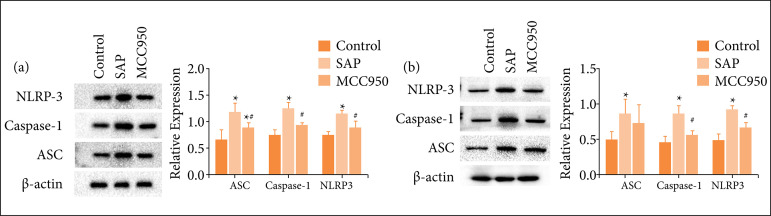
The effect of MCC950 on the expression of NLRP3 inflammasome; **(a)** The expression level of NLRP3 inflammasome in pancreas; **(b)** The expression level of NLRP3 inflammasome (ASC, caspase-1 and NLRP3) in small intestine. The results are expressed as mean ± standard deviation. N = 6; ^*^ p < 0.05 vs. control; ^#^ p < 0.05 vs. SAP.

Compared with the control group, NLRP3 inflammasome (ASC, caspase-1 and NLPR3) expression in pancreatic tissue of the SAP group increased significantly (Fig. 2a), but protein expression of ASC, caspase-1 and NLPR3 in SAP + MCC950 group was lower than those in SAP group (Fig. 2a). The expression of ASC, caspase-1 and NLPR3 in the small intestine of SAP group was significantly higher than those of control group. After intraperitoneal injection of MCC950, the expression of caspase-1 and NLRP3 were down-regulated (Fig. 2b). However, there was no significant difference in the expression of ASC between the two groups (Fig. 2b, p = 0.383).

### MCC950 reduces the severity of pancreatitis in acute pancreatitis mice

**Figure 3:**
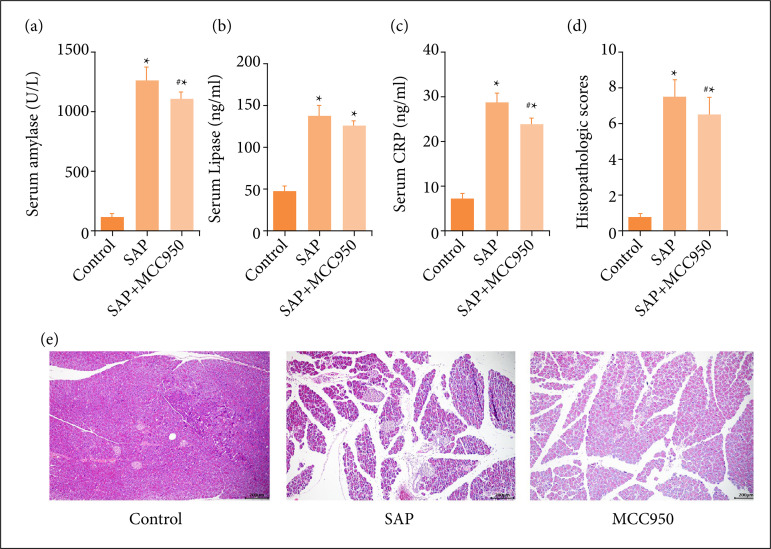
The effect of MCC950 on the expression of NLRP3 inflammasome; **(a)** The expression level of NLRP3 inflammasome in pancreas; **(b)** The expression level of NLRP3 inflammasome (ASC, caspase-1 and NLRP3) in small intestine. The results are expressed as mean ± standard deviation. N = 6; ^*^ p < 0.05 vs. control; ^#^ p < 0.05 vs. SAP.

The levels of serum amylase, lipase and CRP in SAP mice were significantly higher than those in the control group (Fig. 3a–c). Compared with SAP mice, amylase and CRP decreased significantly in the SAP+MCC950 group (Fig 3a,c), but no similar change in serum lipase was observed (Fig. 3b). HE staining was used to observe the pathological changes of pancreatic tissue in each group. The results showed that there were different degrees of edema, hemorrhage, inflammatory cell infiltration and acinar cell necrosis in pancreatic tissue in the SAP group (Fig. 3e). The pancreas pathological score of SAP mice was significantly higher than that in the control group (Fig 3d). Intraperitoneal injection of MCC950 could alleviate pancreatic injury (Fig. 3e), and the pathological injury score was significantly lower than that in the SAP group (Fig. 3d).

### Protective effect of MCC950 on intestinal barrier function

**Figure 4:**
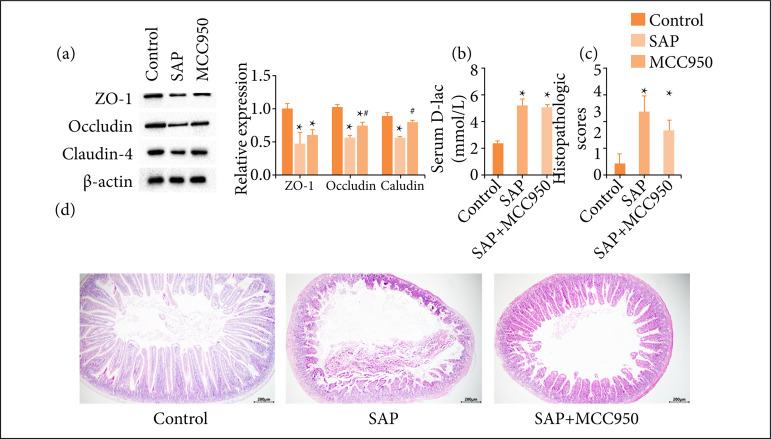
Protective effect of MCC950 on pancreatic injury; **(a)** Amylase; **(b)** Lipase; **(c)** CRP; **(d)** Pancreas pathological score; **(e)** Pancreatic HE staining. N = 6, ^*^ p < 0.01 vs. control; ^#^ p < 0.05 vs. SAP.

The expression level of tight junction proteins (ZO-1, occludin and claudin-4) in the small intestine in each group was analyzed by western blotting. The results showed that claudin-4, occludin, ZO-1 expression in the intestine of SAP mice decreased (Fig. 4a). Compared with SAP mice, the level of intestinal tight junction proteins increased after the intervention with MCC950, and the expression of claudin-4 and occludin were significantly different (Fig. 4a). The level of D-lactic acid in the serum of SAP mice was significantly increased (Fig. 4b), but MCC950 could not reduce the amount of D-lactic acid in serum (Fig. 4b). The pathological changes of the small intestine in each group were observed by HE staining. The intestinal mucosa of SAP mice showed pathological damage, such as shortening, exfoliation, necrosis, and interstitial edema of small intestinal villi. Intraperitoneal injection of MCC950 could alleviate intestinal injury, and the pathological injury score decreased significantly (Fig. 4c,d).

## Discussion

This study emphasizes the inhibitory effect of MCC950 on the production of NLRP3 inflammasome and inflammatory cytokines in SAP mice. The severity of acute pancreatitis and intestinal injury were significantly reduced after treating with MCC950.

The level of inflammatory cytokines can be used to predict the prognosis of SAP^20^^,^ 21. Studies have shown that IL-1β is significantly upregulated in the cerulein-induced acute pancreatitis model, suggesting that inflammatory cytokines are involved in the occurrence and development of pancreatitis^22^. Kaplan *et al*.^23^ found that the IL-1β receptor antagonist (Anakinra) could reduce the severity of acute pancreatitis. In this study, the levels of serum IL-1β and IL-6 in SAP mice were significantly higher than those in the control mice. IL-1β and IL-6 were the end products of NLRP3 inflammasome activation and important mediators of inflammatory cascade in acute pancreatitis^24^^,^ 25. Therefore, whether MCC950 could down-regulate the expression of proinflammatory cytokines was further explore. In this study, the level of serum IL-1β and IL-6 in SAP mice treated with MCC950 were significantly lower than those in SAP mice. Ismael *et al*.^26^ found that MCC950 could improve the neurological impairment of mice with focal cerebral ischemia by inhibiting the expression of IL-1β. S. Li *et al*.^27^ confirmed that MCC950 could inhibit the expression of serum IL-6 and myocardial NLRP3 inflammasome, thus improving the cardiac function of sepsis rats. Therefore, blocking the activation pathway of NLRP3 inflammasome could reduce inflammatory cytokines releasing, such as IL-1β and IL-6, and the intensity of inflammatory response^12^. These results suggest that NLRP3 inflammasome activation is involved in the inflammatory injury of acute pancreatitis.

The activation of NLRP3 inflammasome promotes the initiation of the inflammatory storm, and MCC950 can reduce the expression level of NLRP3 inflammasome^11^^,^ 28. NLRP3 inflammasome acts a principal role in the pathogenesis of various inflammatory diseases^29^. MCC950 is a newly discovered selective NLRP3 inflammasome inhibitor, which can inhibit the activation through two pathways. In the classical pathway, MCC950 inhibits the activation of caspase-1 and the processing of IL-1β. In the non-classical pathway, MCC950 blocks caspase-11-induced NLRP3 activation. In addition, MCC950 can also inhibit the oligomerization of ASC induced by NLPR3, which is a key step in the activation of NLPR3 inflammasome^17^. The activity of NLRP3 inflammasome in SAP mice was positively correlated with the severity of pancreatic injury^3^^,^ 30. This study showed that the expression of NLRP3 inflammasome in pancreatic tissue of SAP mice treated with MCC950 was down-regulated. MCC950 significantly reduced pancreatic pathological damage in SAP mice. Serum amylase and CRP also decreased significantly, indicating that the severity of pancreatitis was reduced. Several studies yielded results similar to this paper. Kim *et al*.^31^ confirmed that fraxinellone could inhibit the infiltration of inflammatory cells in acute pancreatitis mice and reduce the severity of pancreatitis by inhibiting inflammasome activation. Another study established a model of SAP using NLRP3 knockout (NLRP3^(-/-)^) mice and confirmed that NLRP3 deficiency alleviates SAP and pancreatitis-associated lung injury^18^. Therefore, the protective effect of MCC950 on extra-pancreatic organs in SAP mice was further investigated.

Inflammatory cytokine storm in acute pancreatitis often involves the intestinal tract, resulting in damaging the gastrointestinal function^32^^,^ 33. At present, there is no study on how MCC950 affects the damage of small intestinal function in SAP. Therefore, this study further investigated the expression level of NLRP3 inflammasome in the small intestine of SAP mice and the effect of MCC950 on it. The results showed that MCC950 could inhibit the expression of caspase-1 and NLRP3 in the small intestine of SAP mice, but had no significant effect on the expression of ASC. Pan *et al.* 34 found that the inhibitor of NLRP3 activation pathway can reduce the expression of NLRP3 and ASC in the colon. This difference may be due to the different sampling sites of the experiments. In addition, MCC950 could reduce the severity of intestinal injury associated with SAP through histopathological examination. However, in terms of intestinal tight junction proteins, this paper only observed the upregulation of occludin and claudin-4, which may be related to the irreversible damage of intestine during the induction of the SAP model.

## Conclusions

MCC950 could inhibit the expression of NLRP3 inflammasome in the pancreas of acute pancreatitis mice, as well as the expression of caspase-1 in the small intestine and down-regulate the expression of cytokines, thus alleviating pancreatic and intestinal injury. Inhibiting the activation of NLRP3 inflammasome may be an effective strategy for preventing and treating acute pancreatitis. This experiment used a single dose of MCC950 intervention, the best treatment strategy of MCC950 in the treatment of acute pancreatitis still requires further investigation.
